# In Vivo Delivery of *Tinospora cordifolia* Root Extract Preventing Radiation-Induced Dystrophies in Mice Ovaries

**DOI:** 10.1155/2015/346427

**Published:** 2015-08-18

**Authors:** Riddhi Sharma

**Affiliations:** ^1^Amity Education Valley, Kant Kalwar, NH-11C, Jaipur Delhi Highway, Jaipur, Rajasthan 303007, India; ^2^Department of Zoology, University of Rajasthan, JLN Marg, Jaipur, Rajasthan 302004, India

## Abstract

Unconscious and unplanned radiation exposures are a severe threat to gonads particularly ovaries. The present study aims at finding radioprotective effect of *Tinospora cordifolia* (Willd.) Miers root extract (TCRE) in ovaries. Swiss albino mice were divided into four groups: Group 1 served as “normal” and is administered double distilled water and Group 2 is given TCRE with optimum dosage selected as 75 mg/mice. Group 3 serving the purpose of “irradiated control” were exposed to 2.5 Gy gamma radiation. Group 4 (experimental) were administered optimum dosage of TCRE with prior exposure to 2.5 Gy gamma radiation. Follicle cell counts were scored at autopsy intervals of 24 hrs, 3 days, 7 days, 15 days, and 30 days after gamma irradiation. To understand the mechanism of radioprotection, lipid peroxidation (LPO) and glutathione (GSH) levels were also measured in all groups. TCRE supplementation rendered significant protection to ovaries by restoring follicle counts; it also reduced LPO levels and increased GSH levels in ovaries. It implies that TCRE administration protects ovaries against radiation exposure.

## 1. Introduction

In females, miscarriages, sterility, and higher risks of developing various types of cancers are a common outcome of unhealthy and gadget freak lifestyle worldwide. In addition, unplanned/planned or therapeutic radiation exposures can cause permanent sterility in female reproductive organs [1]. Radiation exposures for low doses for longer time periods increase risk of having cancer [2, 3]. If the exposure doses are high it may lead to premature aging and death [4]. Female gonads are highly vulnerable as there is a fixed supply of germ cells endowed at birth; if once lost they can never be replenished [5]. It is reported in literature that radiation exposure increases “redox burden” by producing reactive oxygen species (ROS) manifesting in initiation and progression of cancerous tumor cells [6].

In order to protect the cells from oxidative damage inflicted by radiation, various chemical based radioprotective drugs, namely, WR2721, AET, 2-MPG, and amifostine, were being used. Major handicap of these drugs is that they have inherent toxicity of their own [7, 8]. Chemical radioprotectors were found to interfere with the normal immune-stimulatory and pharmacokinetic mechanism by which cell milieu can self-repair. They have lesser efficacy and generate side effects.

Various plants have been reported to be beneficial for radiation-induced free medical induced conditions [9]. It is implied that plants contain group of compounds that can protect against radiation-induced reactive oxygen species (ROS) and reactive nitrogen species (RNS) mediated damage [10].* Tinospora cordifolia* (Willd.) Miers belonging to family Menispermaceae is acclaimed as divine elixir and extensively used in Ayurveda system of medicine. The plant is rich source of bioactive compounds such as immunologically active arabinogalactan [11], clerodane diterpenes [12], syringing, magnoflorine, cordifoliside A, and tinosporidine [13, 14] functioning as antioxidants by free radical scavenging. The plant is having medicinal properties as antihyperglycemic [15], immune stimulating [16], antioxidant [17], hepatoprotective [18], and antineoplastic [19] which have been assigned to the plant for more than a decade. Radioprotective effect of the plant has also been studied earlier by authors [20, 21]. The present study investigates the radioprotective effect of (TCRE) particularly on ovaries by scoring follicle cell counts and assaying LPO and GSH.

## 2. Materials and Methods

### 2.1. Preparation of TCRE

The aerial roots of* Tinospora cordifolia* (Willd.) Miers were collected from Plant Nursery, University of Rajasthan; afterwards proper identification was made at Herbarium, Department of Botany, University of Rajasthan, and Voucher number RUBL 20132 was collected. The roots (500 g) collected were shade dried and the powdered mixture was refluxed with double distilled water (DDW) and ethanol in ratio of 1 : 1 for 36 hours at 40°C. The liquid extract was cooled and concentrated by evaporating its contents in oven maintained at room temperature. The yield of ethanol : water (1 : 1) mixture or hydroalcoholic extract was 16.55% w/w of crude drug power. The extract was stored at approximately 1–1.5°C temperature. The dose selected as 75 mg/Kg body weight was prepared by redissolving in DDW and administered by oral gavage.

### 2.2. Animals

Swiss albino mice (*Mus musculus*) of BALB “c” strain were maintained as per guidelines set by WHO, Geneva, Switzerland, and INSA (Indian National Science Academy, New Delhi, India). Animals were housed in polypropylene plastic cages in a well-ventilated air cooled room at the temperature of 25 ± 2°C with light and darkness ratio of 14 : 10 hrs. These animals were fed with standard ready-made feed obtained from Ashirwad Industries, Chandigarh, India, and water was provided* ad libitum*. For the experimentation, healthy young-adult female (virgin) mice of 6 weeks of age with a minimum average weight of 23 ± 3 gm were sacrificed. Tetracycline water once a fortnight was given as preventive measures against infections. The Departmental Animal Ethical Committee approved this study.

### 2.3. Irradiation

The cobalt teletherapy unit (ACT-C9) at Cancer Treatment Center, Radiotherapy Department, SMS Medical College & Hospital, Jaipur, was used for irradiation. Unanesthetized animals were restrained in well-ventilated Perspex boxes and underwent whole-body exposure to gamma radiation at the source to surface distance (SSD) of 84.9 to deliver at a dose rate of 1.36 Gy/min.

### 2.4. Processing of Ovaries

The ovaries were quickly taken out and rinsed in chilled 0.15 M Tris-KCl (pH 7.4), blotted, and immediately stored at −80°C till further processing. The homogenates were prepared in cold 0.15 M Tris-KCl buffer (pH 7.4) to yield 10% w/v. The homogenates were centrifuged at 1000 ×g for 10 minutes at 4°C. The resulting supernatant was used for antioxidant enzyme assays.

### 2.5. Histology

In order to score ovarian follicles, ovaries from mice were dissected free of fat and mesentery. Intact ovaries were placed in Bouin's fixative for histology within 30 min of dissection. Fixed tissues were embedded in paraffin wax, serially sectioned at 5 *μ*, and stained with haematoxylin and eosin. The tissue sections were numbered and then examined for the presence of follicles.

### 2.6. Classification of Ovarian Follicles

In the present study follicle cell population were broadly divided into 3 subheads: small, medium, and large follicles were labeled as categories A, B, and C, respectively. Category “A” corresponds to primordial follicles with up to one layer and two layers of flattened and cuboidal granulosa cells. Category “B” comprises preantral follicles with three or more layers of granulosa cells and no antrum. Category “C” includes antral follicles with antral cavity and corpora lutea.

### 2.7. Scoring of Ovarian Follicles

The total number of category “A” follicles in each ovary was estimated by multiplying the number of follicles counted by five. Category A and B follicles were counted in the section where the nucleolus was seen within the nucleus of the oocyte to avoid counting them more than once. Corpora lutea were scored at their maximum diameter. The number of corpora lutea corresponded closely to the number of ovulated oocytes found at the periphery of the grafts.

### 2.8. A Dose Tolerance Study to Select the Optimum Dose of TCRE against Irradiation

To select the optimum dose of TCRE for radioprotection, the mice were divided into two groups, namely, control (DDW + 2.5 Gy) and experimental group (TCRE + 2.5 Gy). The experimental group received 25, 50, 75, 100, 150, and 200 mg/kg/body wt/day of TCRE orally, before exposure to 10 Gy of gamma radiation. A dose of 75 mg/kg/body wt/day TCRE was found to be the optimum radioprotective dose and therefore further experiments were carried out using this dose.

### 2.9. The LD_50/30_ and Dose Reduction Factor

The efficacy of any protective agent is evaluated by the determination of its dose reduction factor (DRF). The DRF of TCRE based on LD_50/30_ survival experiment was calculated after irradiating a large number of Swiss albino mice to different doses of gamma rays in the presence (experimental) or absence (control) of TCRE. The percentage of mice surviving at each radiation dose till 30 days following such exposures was used to construct survival dose response curves. Regression analysis was done to obtain LD_50/30_, and dose reduction factor was computed as

(1) $$\begin{array}{c} \text{DRF}=\frac{{\text{LD}}_{50/30}\text{ of Experimental Animals}}{{\text{LD}}_{50/30}\text{ of Control Animals}}. \end{array}$$

On the basis of previous experiments, the dose reduction factor of TCRE against radiation treatment was calculated on the basis of the survival experiment and was measured as 1.68.

### 2.10. Modification of Radiation Response

The animals selected for the study were divided into four groups: Group 1: mice of this group were administered orally double distilled water (volume equivalent to TCRE) and considered as normal. Group 2: mice of this group received the optimum dose of TCRE (75 mg/kg/body wt/day) by gavage, daily for 5 consecutive days, without exposure to radiation. This group served as drug or plant extract alone treated group. Group 3: mice of this group were given DDW (equal volume of TCRE), orally and daily for 5 consecutive days, followed by whole-body exposure to 2.5, 5.0, and 7.5 on 5th day of DDW administration. It served as irradiated control. Group 4: mice of this group were administered optimum dose of TCRE (i.e., 75 mg/kg/body wt/day) orally for 5 consecutive days and exposed to 2.5 Gy gamma radiation on the last day after half an hour of TCRE administration (5th day). This group served as irradiated experimental group.

### 2.11. Lipid Peroxidation (LPO) Assay

LPO was determined as the concentration of MDA according to the method of Ohkawa et al. [22]. Briefly 25 *μ*L of ovarian homogenates (10% w/v) was taken in test tubes containing 370 *μ*L of 20% (v/v) glacial acetic acid (pH 3.4), 50 *μ*L of 8% (w/v) sodium dodecyl sulphate, 370 *μ*L of 0.85% thiobarbituric acid (TBA), and 170 *μ*L of double distilled water. The test tubes were incubated at 80°C for 60 min. Afterwards test tubes were centrifuged at 2500 ×g for 15 minutes. The amount of malondialdehyde (MDA) formed was measured spectrophotometrically at 532 nm.

### 2.12. Glutathione Assay

Reduced glutathione was estimated as total nonprotein sulfhydryl group by the method described by Moron et al. [23]. Homogenates were immediately precipitated with 25 *μ*L of 25% trichloroacetic acid and the precipitate was removed after centrifugation. Free SH groups were assayed in a total 750 *μ*L volume by adding 500 *μ*L of 0.6 M 5,5 dithiobis-2-nitrobenzoic acid (DTNB) prepared in 0.2 M sodium phosphate buffer (pH 8.0). The supernatant was spectrophotometrically measured at 412 nm.

### 2.13. Statistical Analysis

The results for all the groups at various autopsy intervals were expressed as mean ± standard error (S.E.). Student's *t*-test was used to find the significant difference of sample drawn from experimental group (Group 4) from respective control (Group 3), by the method of Daly and Bourke [24]. The significance level was set at different levels as *p* ≤ 0.05, *p* ≤ 0.01, and *p* ≤ 0.001.

## 3. Results

A dose selection for TCRE was done on the basis of a drug tolerance study. For this purpose, various doses of TCRE extract (100, 200, 400, 800, 1000, 1500, and 2000 mg/kg/body wt) were tested for their tolerance (once a day for 5 consecutive days) in Swiss albino mice. One hour after the last administration of TCRE, mice were exposed to 10 Gy gamma irradiation. All these animals were then observed for 30 days for scoring signs of radiation sickness or mortality. Body weights of the animals were also measured. Thus, the optimum tolerated dose of TCRE (75 mg/kg/body wt) was determined and used for further detailed experimentation [25].

The efficacy of any protective agent is evaluated by the determination of its dose reduction factor (DRF). The dose reduction factor of* Tinospora cordifolia *against radiation treatment was calculated on the basis of the survival experiment and was measured as 1.68 [25].

**Figure 1:**
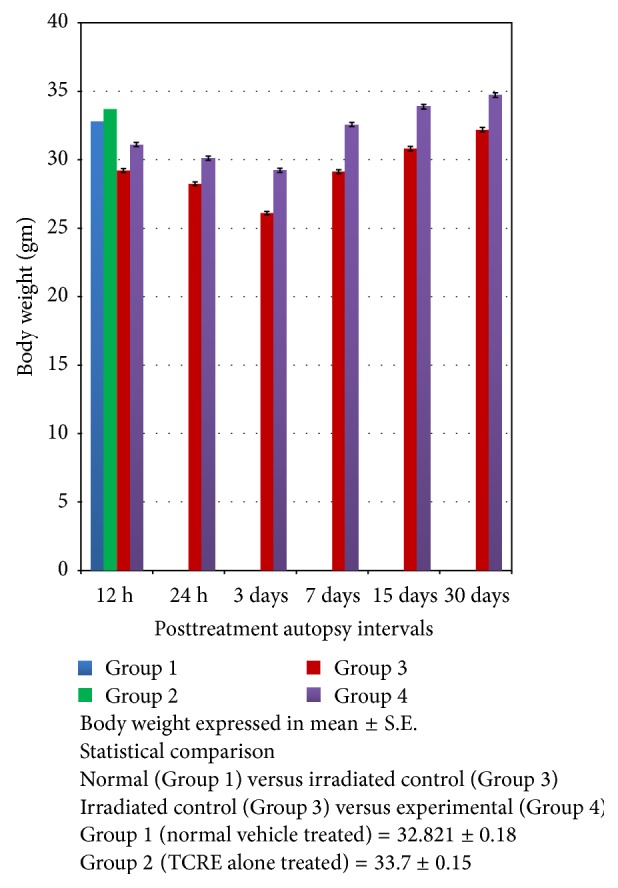
Variations in the body weight (gm) of mice after exposure to different doses of gamma radiation with (experimental) or without (irradiated control) TCRE.

Group 3 evidenced 10.94% of normal reduction of body weight at 12 hrs. Recovery starts after 7th day postautopsy interval. In the TCRE administered group [4] in spite of irradiation the body weights were close to normal (Group 1) values (Figure 1).

**Figure 2:**
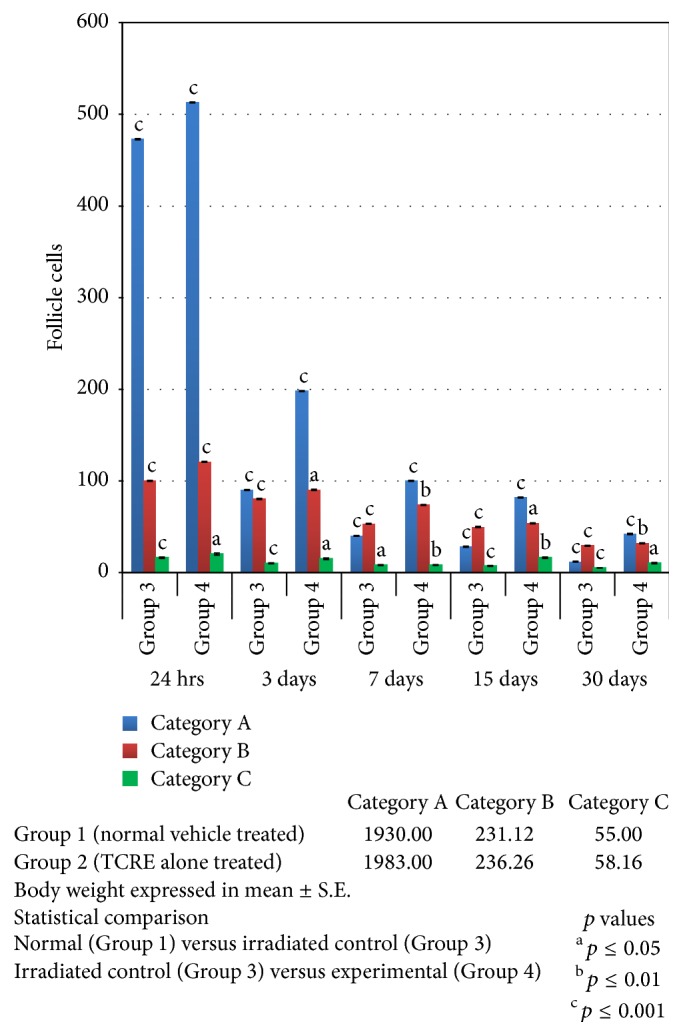
Follicle cell population at various intervals after exposure to 2.5 Gy gamma radiation with (experimental) or without (irradiated control) TCRE.

In Group 3 there is “*p* ≤ 0.001” significant decrease in follicle cell population comparable to normal (Group 1) for category “A.” Similarly, follicle cell population continues to decrease (*p* ≤ 0.001) till 30th day in Group 3 for categories “B” and “C.” In TCRE treated animals (Group 4) for category “A,” there is 8.47% (*p* ≤ 0.001) increase in number of follicles comparable to Group 3 at 24 hrs. Similar restorative behavior is being experienced by categories “B” and “C” as shown in Figure 2.

**Figure 3:**
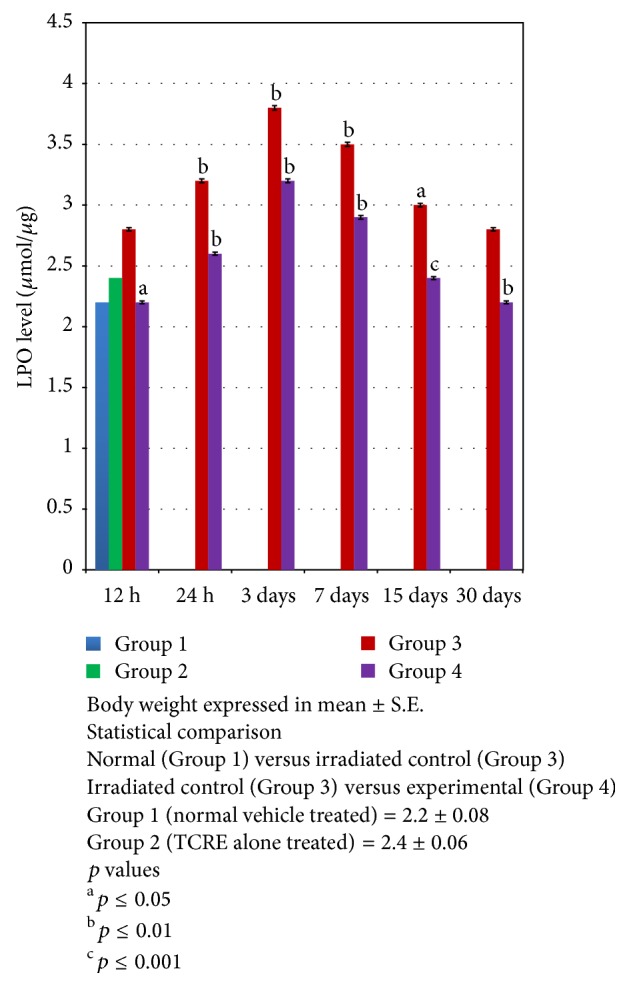
Variation in lipid peroxidation (LPO) level (*μ*mol/*μ*g) in ovaries of mice exposed to different doses of gamma radiation with (experimental) or without (irradiated control) TCRE.

After radiation exposure, a significant (*p* ≤ 0.001) increased level of lipid peroxidation was evident in the ovaries up to day 15 (3.0 ± 0.05) of Group 3 and up to day 3 (3.2 ± 0.01) in Group 4. Thereafter a notable depletion in LPO was recorded at the remaining interval in Group 3 as well as in Group 4. However the observed values were higher in irradiated controls as compared to experimental group and at the last autopsy interval these were found as 21.42% higher than normal (Figure 3).

**Figure 4:**
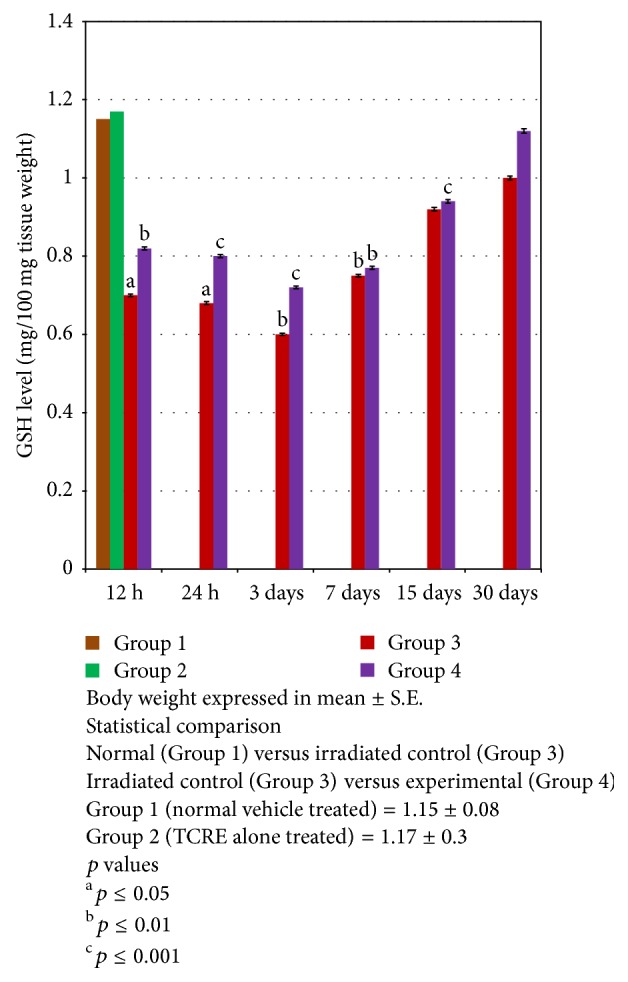
Variations in glutathione (GSH) level (mg/100 mg tissue weight) in ovaries of mice exposed to different doses of gamma radiation with (experimental) or without (irradiated control) TCRE.

A steady decreasing pattern was followed by ovarian glutathione level up to day 7 in both irradiated control (Group 3) and experimental group (Group 4) (0.75 ± 0.11, *p* ≤ 0.01 and 0.77 ± 0.3, *p* ≤ 0.01, resp.). However, such extent of decrease was comparably lower in the experimental group than the respective irradiated control at all autopsy intervals. Thereafter, a significant elevation was recorded at the consecutive intervals, but the level was significantly (*p* < 0.01) lower in Group 4 (Figure 4).

## 4. Discussion

The vulnerability to reproductive function following radiotherapy is of paramount importance [26]. The results decocted in the present study did not mirror any evident toxic effects as with intake of TCRE. Ionizing radiations have adverse effects on gonadal function at all ages. In the present study, irradiation to 2.5 Gy results in radiation sickness within 1–3 days after exposure. Group 3 animals evidenced weight loss due to impaired functioning of GI tract [27–29]. Radiations elicit particular syndromes, that is, HPS, GIS, and NVS [30]. It is also evident in the study that prevenient administration of TCRE in Group 4 does not only discourage the onset of radiation sickness but also allay its magnitude appreciably. As studied by various authors [31, 32] various bioactive ingredients of* Tinospora cordifolia* by cumulative mechanisms, namely, metal chelation, free radical scavenging, and cellular DNA repair, render protection against radiation-induced weight loss. As per previous reports the estimated dose at which half of the follicles are lost in humans is 4 Gy [33].

Qualitative and quantitative assessment of ovary has profound utilities to elicit degree of ovarian toxicity and their site of damage and assess infliction to primordial follicles and ovarian lesions [34–37]. The complement of developing follicles within ovaries originates from immature nongrowing fixed stock of primordial follicles. Coordinated entry of these follicles into the growth phase controls the rate at which follicular reserve is depleted. In the present study, the significant decrease in the ovarian cell population in Group 3 is as a result of DNA damage resulting from ionizing radiation [38]. From the previously mentioned results it was obvious that the irradiation caused damage in the genomic DNA which appeared as a decrease in the number of the amplified DNA fragments in the irradiated group as compared to the control group. The disappearance of normal bands may be related to events such as DNA damage (e.g., single- and double-strand breaks, modified bases, abasic sites, oxidized bases, bulky adducts, and DNA protein cross-links), point mutations, and/or complex chromosomal rearrangements induced by genotoxins [39–41]. It has been long recognized that the damaging effect of ionizing radiation is brought about by direct DNA ionization as well as indirectly through ROS production. As a consequence, thiols like GSH and other antioxidant enzymes compete with this oxidation, chemically reduce the free radicals, and repair the damage [42]. The protective role of TCRE may be due to the higher content of secondary metabolites including anthraquinones, terpenoids, and saponins which were present in many extracts in addition to phenolics which have the ability to scavenge free radical and enhance the DNA repair system or DNA synthesis. The intake of* Tinospora cordifolia* can reduce cancer risk in humans via a decrease in DNA damage.

Radiotherapy has radically increased long-term survival of young female cancer patients, but major side effects of this treatment are ovarian failure and infertility [43]. The deleterious effects of ionizing radiation on biological systems are mainly mediated through the generation of reactive oxygen species (ROS) in cells as a result of water radiolysis [44]. Low levels of ROS are important signal in regulation of physiological functions in female reproduction, including folliculogenesis, steroidogenesis, corpus luteum function, and luteolysis [45]; however, increasing ROS production has a key role in pathological processes in female reproduction [46, 47]. Additionally, apoptosis is an essential component of ovarian function and development, which is responsible for oocyte loss [48], and it can be further induced by radiation [49]. Gamma irradiation causes either cell cycle block or apoptosis through the p53-mediated p21 activation [50]. p21 is an inhibitor of cyclin-dependent kinases and may also directly inhibit PCNA in DNA replication [51].

The ovary histopathological examination in the present study showed that there were no abnormal histological alterations in the groups treated with TCRE alone as compared to the control (Group 3). Conversely, in irradiated ovaries, there were various numbers of corpora lutea replacing the ovary tissues and the blood vessels appeared dilated and filled with blood. There were areas of hemorrhages that were seen in the lumen of corpus luteum. The present findings are in agreement with Said et al. [27] who found that ovaries two weeks after irradiation showed more corpus luteum formation with appearance of ovarian cysts. It has been long recognized that the damaging effect of ionizing radiation is brought about by direct DNA ionization as well as indirectly through ROS production. In the present study the levels of LPO were high in irradiated Group 3. As a consequence, thiols like GSH and other antioxidant enzymes compete with this oxidation, chemically reduce the free radicals, and repair the damage [42]. So it was studied that the treatment with* Tinospora cordifolia* root extracts before irradiation minimized the damages caused by irradiation. Interestingly, treatment of irradiated females with TCRE before irradiation preserved the ovarian tissue from radiation-induced damages. The protective effect of TCRE against the radiation-induced damages might be due to their ability to block oxidative stress and lipid peroxidation of membranes.

TCRE possesses one of the highest concentrations of antioxidant vitamins and is known to protect cells against oxidative stress, in particular DNA damage by increasing GSH levels as was evident in Group 4 [52, 53].

An immune stimulating compound *α*-d-glucan (RR1) is composed of (1 → 4) linked backbone and (1 → 6) linked branches isolated from* Tinospora cordifolia* [16].

Active bioactive compounds like sesquiterpene, tinocordifolin, glycoside, tinocordifolioside, and arabinogalactan are present in good amount in* Tinospora cordifolia*. Alkaloids such as berberine and magnoflorine are the major chemical compounds isolated from the aerial roots of* T. cordifolia* [54]. These active compounds are rendering the extract its antioxidant status [55].

## 5. Conclusions

Results of the present study suggest that TCRE administration provides protection against radiation-induced ovary damage. However, further studies are required to determine the exact component of TCRE responsible for the observed effects and the mechanism involved.
